# Bone Regeneration with Dental Pulp Stem Cells in an Experimental Model

**DOI:** 10.3390/jpm14111075

**Published:** 2024-10-25

**Authors:** Haifa Hamad-Alrashid, Sandra Muntión, Fermín Sánchez-Guijo, Javier Borrajo-Sánchez, Felipe Parreño-Manchado, M. Begoña García-Cenador, F. Javier García-Criado

**Affiliations:** 1 Doctoral School “Studii Salamantini”, University of Salamanca, 37008 Salamanca, Spain; hrashed94@usal.es; 2 Biomedical Research Institute (IBSAL), 37007 Salamanca, Spain; smuntion@usal.es (S.M.); fjgc@usal.es (F.J.G.-C.); 3 Regenerative Medicine and Cellular Therapy Network Center of Castilla y León, 37007 Salamanca, Spain; ferminsg@usal.es; 4 Hematology Department, University Hospital of Salamanca, 37007 Salamanca, Spain; 5 Department of Medicine, Faculty of Medicine, University of Salamanca, 37007 Salamanca, Spain; 6 Department of Biomedical and Diagnostic Sciences, Faculty of Medicine, University of Salamanca, 37007 Salamanca, Spain; borrajo@usal.es; 7 Department of Surgery, Faculty of Medicine, University of Salamanca, 37007 Salamanca, Spain; fparreno@usal.es; 8 Coordinator of the Esophagogastric Surgery and Obesity Unit, University Hospital of Salamanca, 37007 Salamanca, Spain

**Keywords:** DP-MSC, biomaterials, bone defect, osteogenesis, bone regeneration, angiogenesis, bioengineering, dental pulp, stem cells, bone graft

## Abstract

Background/Objectives: The therapeutic approach to bone mass loss and bone’s limited self-regeneration is a major focus of research, emphasizing new biomaterials and cell therapy. Tissue bioengineering emerges as a potential alternative to conventional treatments. In this study, an experimental model of a critical bone lesion in rats was used to investigate bone regeneration by treating the defect with biomaterials Evolution^®^ and Gen-Os^®^ (OsteoBiol^®^, Turín, Italy), with or without mesenchymal stromal cells from dental pulp (DP-MSCs). Methods: Forty-six adult male Wistar rats were subjected to a 5-mm critical bone defect in the right mandible, which does not regenerate without intervention. The rats were randomly assigned to a Simulated Group, Control Group, or two Study Groups (using Evolution^®^, Gen-Os^®^, and DP-MSCs). The specimens were euthanized at three or six months, and radiological, histological, and ELISA tests were conducted to assess bone regeneration. Results: The radiological results showed that the DP-MSC group achieved uniform radiopacity and continuity in the bone edge, with near-complete structural defect restitution. Histologically, full bone regeneration was observed, with well-organized, vascularized lamellar bone and no lesion edges. These findings were supported by increases in endoglin, transforming growth factor-beta 1 (TGF-β1), protocollagen, parathormone, and calcitonin, indicating a conducive environment for bone regeneration. Conclusions: The use of DP-MSCs combined with biomaterials with appropriate three-dimensional matrices is a promising therapeutic option for further exploration.

## 1. Introduction

The number of serious bone lesions has increased sharply due to, among other reasons, the localized and generalized loss of bone mass. Numerous studies report that many pathologies and medications are linked to the loss of bone mass [1,2,3], which together with a more sedentary lifestyle impacts negatively on bone metabolism.

The therapeutic need to treat bone lesions and/or defects has required the use of pharmacological and surgical treatments that need to be improved [4]. Faced with the scarce bioavailability of autologous grafts, the low osteoinductive capability of xenografts, and the rejection of allografts, biomaterials are the ideal option. Cells, three-dimensional supports or matrices, and growth factors are key elements [5], in addition to an appropriate physical and chemical environment. Tissue engineering and cell therapies thus provide alternatives for prompting and promoting bone regeneration, focusing on bespoke and precision regenerative medicine and reducing the morbidity of other treatments. The availability of biomaterials with a suitable physical structure enables cell adhesion and proliferation, playing a major role in the repair and regeneration of bone tissue [6,7,8], with a crucial aspect being our advanced understanding of stem cells [9,10,11] and the growth factors that favor differentiation and accelerate these processes [12,13].

The therapeutic potential of mesenchymal stem cells (MSCs) in regenerative medicine is well known, based on such characteristics as self-renewal and differentiation into diverse lineages for repairing damaged tissues and modulating the immune system and inflammation. MSCs have been obtained from a variety of human tissues, including adipose tissue, bone marrow, umbilical cord, placenta, and dental pulp [14,15]. In particular, MSCs from dental tissue are being widely used as a tool in tissue engineering due to their ease of collection, rapid growth, and the amount of factors secreted by these cells [16]. Dental pulp was the dental tissue in which MSCs were first observed [17].

DP-MSCs have similar characteristics to BM-MSCs in terms of bone regeneration and the gene expression involved in osteogenesis and mineralization [18,19]. In addition, DP-MSCs have been used for treating neurological, circulatory, immune, and oral diseases due to their immunomodulatory, anti-inflammatory, antiapoptotic, and low immunogenicity properties [20]. Several biomaterials have been combined with DP-MSCs, revealing their biocompatibility, viability, and bone regeneration in both in vitro and in vivo studies [21]. Some studies have reported an increase in bone regeneration in animals treated with biomaterials plus DP-MSC compared to those treated with biomaterials alone [22,23,24].

This study evaluates an experimental model involving the bone regeneration of a rat mandibular defect using stems cells of a mesenchymal origin obtained from pulp material with the view of finding a suitable biological substitute and increasing the potential of the MSCs using biological supports with a high concentration of osteogenic factors.

## 2. Materials and Methods

### 2.1. Experimental Design, Specimens, and Groups

The sample consisted of 46 WISTAR Han™ (RccHan™:WIST) adult male rats (8 months—350 g) supplied by Charles River Laboratories, (Wilmington, MA 01887, USA). All the experiments were conducted following the guidelines established by Spain’s Royal Decree 53/013 (approval by the Ethics Committee at Salamanca University, filed under number 361).

**Figure 1:**
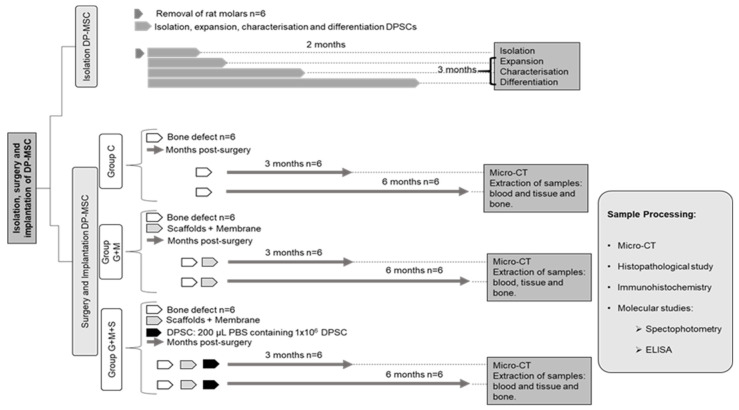
Study experimental design. Group C: rats with bone defect. Group G + M: rats with bone defect plus scaffolds and membrane. Group G + M + S: rats with bone defect plus scaffolds, membrane, and MSCs from dental pulp.

Figure 1 describes the experimental design of the study.

The molars of rats were used for the extraction of DP-MSCs (two pulps per extraction), as described in due course (*n* = 6).

The 40 remaining rats were distributed into the following groups:

- Sham (Sham): Animals were subjected to the same anesthetic/surgical technique as the other groups except for the bone lesion and its treatment, as indicated below. The specimens remained caged for three months, whereupon the samples were gathered, and they were euthanized (*n* = 4).
- Control (C): The bone lesion was induced but not treated, as indicated below. The specimens remained caged for three or six months (*n* = 6 in each case), whereupon the samples were gathered, and they were euthanized (*n* = 12).
- Gen-Os^®^ + Evolution^®^ (G + M): The bone lesion was induced and then treated with the substitute bone biomaterial and covered with resorbable membrane, as indicated below. The specimens remained caged for three or six months (*n* = 6 in each case), whereupon the samples were gathered, and they were euthanized (*n* = 12).
- Gen-Os^®^ + Evolution^®^ + DP-MSC (G + M + S): The bone lesion was induced and then treated with the substitute bone biomaterial plus DP-MSCs and covered with resorbable membrane, as indicated below. The specimens remained caged for three or six months (*n* = 6 in each case), whereupon the samples were gathered, and they were euthanized (*n* = 12).

### 2.2. Biomaterials

The Gen-Os^®^ (OsteoBiol^®^, Torino, Italy) 250–1000 µm granulated bone substitute used was of biological origin (pig) and was based on hydroxyapatite (HA) and type I collagen. Its composition is 80% spongy bone and 20% cortical bone with high osteoconductivity. This three-dimensional matrix provided support for the MSCs from the dental pulp.

The Evolution^®^ (OsteoBiol^®^, Italy) resorbable membrane used involved dense collagen fibers that covered the induced bone defect.

### 2.3. Obtaining the DP-MSCs from Pulp Tissue

Rat molars were extracted under general intraperitoneal anesthesia, using a combination of ketamine hydrochloride (2 mg/kg, Ketolar, Pfizer Inc., New York, NY, USA), diazepam (10 mg/kg, Valium, F. Hoffmann-La Roche Ltd Basel, Basel-Stadt, Switzerland), and atropine (1 mg/kg, Atropine, B. Braun Melsungen AG Melsungen, Hesse, Germany). Following the procedure, the specimens were euthanized by intraperitoneal administration of sodium pentobarbital (Dolethal 100 mL, Vetoquinol S.A. Lure, Bourgogne-Franche-Comté, France) at a dose of 100–150 mg/kg of body weight. This dose, approximately three times the standard anesthetic dose, ensured rapid and painless euthanasia. The entire process was conducted in accordance with the protocols established by the Animal Experimentation Service (SEA) of the University of Salamanca, ensuring the highest standards of animal welfare and minimizing the suffering of the experimental subjects.

The molars were then crushed with gouge forceps, obtaining pulp tissue with a Hemingway^®^ bone curette (Model 1145/0, Integra LifeSciences Corporation, Princeton, NJ, USA). The samples were placed in a falcon tube (50 mL, Corning Incorporated Corning, New York, NY, USA) with 30 mL of DMEM (Dulbecco’s Modified Eagle Medium^®^, Thermo Fisher Scientific, Waltham, MA, USA), supplemented with penicillin (100 U/mL, Sigma-Aldrich/Merck, St. Louis, MO, USA), streptomycin (100 μg/mL, Sigma-Aldrich/Merck, St. Louis, MO, USA), and fetal bovine serum (FBS) at 10% (Gibco, Thermo Fisher Scientific, Waltham, MA, USA), and cold stored for a maximum of six hours.

For enzymatic disaggregation, the tissue was incubated for 70 min at 37 °C and shaken with collagenase I (at 0.2%) (Worthington Biochemicals Corporation^®^, Lakewood, NJ, USA) and dispase II (at 0.4%) (Sanko Junyaku Co. Ltd., Tokyo, Japan). The tissue was centrifuged for 10 min at 1200 rpm, and the sediment was spread on 9 cm^2^ plates and incubated at 37 °C.

Cell culture and growth was performed by incubating the cells at 37 °C in a humid environment with 5% CO_2_ in DMEM (Dulbecco’s Modified Eagle Medium^®^), supplemented with FBS (10%) (Equitech-Bio Inc., Kerrville, TX, USA) and with penicillin (100 U/mL, Sigma-Aldrich/Merck, St. Louis, MO, USA) and streptomycin (100 μg/mL, Sigma-Aldrich/Merck, St. Louis, MO, USA). The culture medium was replaced twice per week, and any unattached cells were discarded. The culture was maintained until a cell confluence of at least 80–90% was reached, whereupon the culture medium was eliminated, washed with sterile phosphate buffered saline—PBS (Gibco/Thermo Fisher Scientific, Waltham, MA, USA) and incubated for five minutes at 37 °C with trypsin 1 (0.05%) (Gibco/Thermo Fisher Scientific, Waltham, MA, USA). Trypsin 1 (Gibco/Thermo Fisher Scientific, Waltham, MA, USA), was neutralized before the addition of a culture medium for MSCs. Cellularity was increased by again seeding a concentration of 5000 cells/cm^2^ in larger beakers, up to passage 10 (S10).

Cells were isolated for their immunophenotypic characterization and induction to differentiation in several steps (1, 3, 6, and 10) to verify their osteoblastic and adipogenic capacity. Three parallel samples were cultivated under the same conditions (at 37 °C, 5% CO_2_, and >90% humidity): one for osteoblast differentiation and the other as control and the last for adipogenic differentiation.

The medium for osteogenic differentiation (NH OsteoDiff Medium, Miltenyi Biotec, Bergisch Gladbach, North Rhine-Westphalia, Germany) contained β-glycerol-phosphate, ascorbic acid-2-phosphate, dexamethasone, and FBS. The cultures were maintained for 10 days, and the medium was changed every 3–4 days. They were then washed with cold PBS and fixed with 70% ethanol fraction (Merck KGaA, Darmstadt, Germany) for 10 minutes. Osteoblast differentiation was confirmed by observing alkaline phosphatase activity by staining for 20–30 min with NBT/BCIP (Nitroblue tetrazolium chloride 5-bromo-4-chloro-3-indolyl-phosphate, Roche, Basel, Switzerland), and contrasting for two minutes with hematoxylin (1 mL) (Merck KGaA, Darmstadt, Germany).

Adipogenic Differentiation: A process similar to the previous one was carried out. Upon reaching confluence, one of the flasks was incubated with differentiation medium for adipocytes (NH Adipodiff Medium, Miltenyi Biotec, Bergisch Gladbach, Germany) formed by DMEM supplemented with 1% penicillin–streptomycin, 10% fetal serum, 10% horse serum, and insulin (Sigma-Aldrich/Merck, St. Louis, MO, USA), at a final concentration of 100 ng/mL, and the other bottle was incubated with mesenchymal cell expansion medium and served as a negative control. Both remained under the same conditions of humidity, temperature, and CO_2_ concentration for 21 days. To verify the existence of cytoplasmic lipid accumulations, O-oil red staining was used (Oil-Red_O solution, Certistain^®^ Merck KGaA, Darmstadt, Germany). For this, the differentiation and expansion media were removed, and the samples were fixed with 3.7% formaldehyde (Merck KGaA, Darmstadt, Germany) for 2 min. Then, 1 ml of Oil-red-O solution was added and incubated for 1 h at room temperature under slow stirring.

### 2.4. Surgical Procedure

All the surgical procedures were performed under strictly sterile conditions in a laminar flow cabinet.

Rats were anesthetized by intraperitoneal injection of ketamine hydrochloride (2 mg/kg, Ketolar, Pfizer Inc., New York, NY, USA) combined with diazepam (10 mg/kg, Valium, F. Hoffmann-La Roche Ltd Basel, Basel-Stadt, Switzerland) and atropine (1 mg/kg, Atropine, B. Braun Melsungen AG Melsungen, Hesse, Germany). The surgical area was shaved, and an antiseptic solution of povidone–iodine (Betadine, Viatris Inc., Canonsburg, PA, USA) was applied.

**Figure 2:**
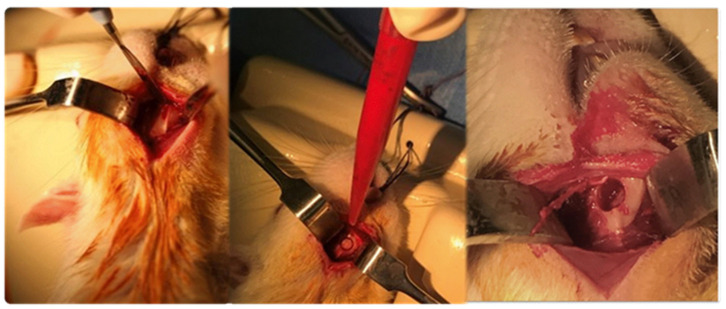
Access to insertion crest. Circular osteotomy with trephine drill.

A 15-mm longitudinal incision was made 2 mm above the lower edge of the mandibular body, providing submandibular entry and access to the ascendant branch and angle of the right jaw, where a circular bone lesion with a 5-mm diameter was made with an electric micromotor and 5-mm trephine drill, which is considered to be a critical bone defect (Figure 2). The bone defect was subsequently covered as described for each group and the incisions were closed in layers. The specimens were caged in a state of good health until three or six months had elapsed after surgery, with direct ad libitum access to food and water.

The post-surgical clinical monitoring involved assessing the specimens’ overall condition, the appearance of the wound and surgical area, bleeding or discharge from the wound, rejection of the biomaterials, and any degenerative changes due to the dental lesion.

Once the monitoring period had ended, the next step involved gathering the samples and euthanizing the specimens:

1. Total blood was collected by aortic puncture, centrifuged (20 min at 4500 rpm and 4 °C), plasma was extracted, aliquoted, and frozen at −80 °C.
2. Perilesional tissue (bone and muscle) was placed in liquid nitrogen and stored at −80 °C.
3. Affected mandibulae were removed in bloc, including removal of the tissue on the bone surface, with some being immersed in formaldehyde 3.7–4.0% *w*/*v* buffered to pH = 7, and others were stored at −80 °C.

### 2.5. Verification of Cell Viability

The DP-MSCs inserted in the biomaterial were viable, with a small volume of the implanted cells being retained and returned to the laboratory for re-seeding.

### 2.6. Macroscopic Study

Prior to euthanizing, a descriptive assessment was made of the following: anatomic and tissue formation; signs of infection; existence of fractures; presence or disappearance of biomaterials; degenerative dental transformations; bone sequestrations; surface morphology; and solidity of the defect.

### 2.7. Scanning with Micro-Computed Tomography (Micro-CT)

At the end of the 3- and 6-month follow-up periods, and prior to euthanasia, two specimens from each group underwent radiographic evaluation using micro-computed tomography (Micro-CT) with SuperArgus equipment from SEDECAL Medical Systems, Madrid, Spain.

The evaluation addressed the regeneration of the defect in the four groups and the different timeframes, together with the changes in the morphology of the biomaterial following its implanting. It also considered the biomaterial’s loss of homogeneity over the study period as a process of integration in the host bone and remodeling. Bone trabeculae were also detected within the biomaterial. The biomaterial’s dispersion within the medullary cavity was studied, together with the appearance on the periphery of the implanted area of bone trabeculae forming “bridges” between the biomaterial and the adjacent cortical bone.

### 2.8. Histomorphometric Evaluation

The hemimandibles were obtained and fixed in 4% formalin for 48 h before being immersed in 10% EDTA for 30 days. The bone tissues were then embedded, and the paraffin blocks were cut into 4-μm sections. The sections from each sample were stained with hematoxylin–eosin (H–E) for their comparative histological study with optical microscopy (Olympus, Tokyo, Japan).

### 2.9. ELISA

This technique was used to determine the concentrations of calciotropic hormones such as calcitonin (CT) and parathormone (PTH), growth factor TGF-β1, levels of type I procollagen, and endoglin expression in the serum of the rats in each group. This involved commercial kits, and the procedures employed followed the manufacturer’s instructions. Parathyroid Hormone (PTH): Enzyme-linked Immunosorbent Assay kit ABK1-E1599. Calcitonin: Enzyme-linked Immunosorbent Assay kit ABK1-E1354. Collagen Type 1 Alpha 2 (COL1α2): Enzyme-linked Immunosorbent Assay kit ABK1-744. transforming growth factor beta 1 (TGF-β1): Enzyme-linked Immunosorbent Assay kit ABK1-E1239. Endoglin (ENG): Enzyme-linked Immunosorbent Assay kit ABK1-E2467.

### 2.10. Statistical Study

The statistical study of the quantitative results was conducted using one-way analysis of variance (ANOVA). As the data did not fit the normal distribution, we applied the Kruskal–Wallis Z-value multiple comparison (Dunn’s Test) with Regular Test or Bonferroni Test. The quantitative data have been represented as X ± SD (mean ± standard deviation). A value of *p* < 0.05 was accepted as a significant result. The statistical software used was NCSS 2007 and Gess 2006—version: 07.1.21—which was released on 1 June 2011 (Dr. Jerry L. Hintze, Kaysville, UT, USA).

## 3. Results

### 3.1. Obtaining Dental Pulp Mesenchymal Stem Cells (DP-MSCs)

**Figure 3:**
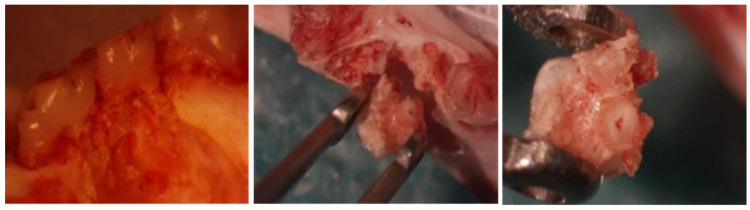
Extraction of pulp.

The pulps were successfully obtained from all the molar chambers (Figure 3).

#### 3.1.1. Cell Culture and Expansion

**Figure 4:**
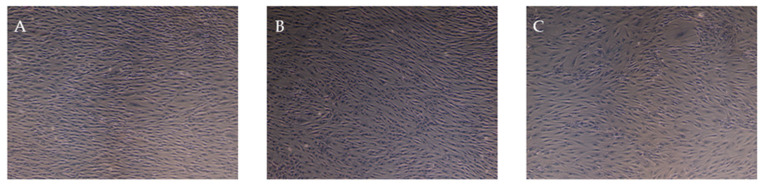
Brightfield microscopy of rat DP-MSCs at different culture passages: (**A**) at passage 4, (**B**) at passage 7, and (**C**) at passage 10. Image magnitude 10×.

The results show that MSCs can be isolated by enzymatic digestion from dental pulp tissue. All DP-MSCs obtained from the culture showed fibroblastic morphology and met the criteria established by the International Society of Cell Therapy (Figure 4).

#### 3.1.2. Characterization: Flow Cytometry

From each animal, the cultures were investigated for the percentage of MSCs by flow cytometry, using the following panel: CD49e PE (HMa5-1, BioLegend^®^ cat. 103905), 7-AAD (BioLegend^®^ cat. 420403), CD11b/c PE-Cy7 (OX-42, BioLegend^®^ cat. 201817), CD29 APC (HMb1-1, Miltenyi Biotec cat. 130-123-829), CD90-1 APC-/Fire™ 750 (Thy1.1, BioLegend^®^ cat. 202543), and CD45 Pacific Blue™ (OX-1, BioLegend^®^ cat. 202225). The samples were acquired in the BD FACSCanto™ II System, and the data were analyzed using FlowJo™ Software (v. 10, ©Becton Dickinson, San Jose, CA 95131, USA).

**Figure 5:**
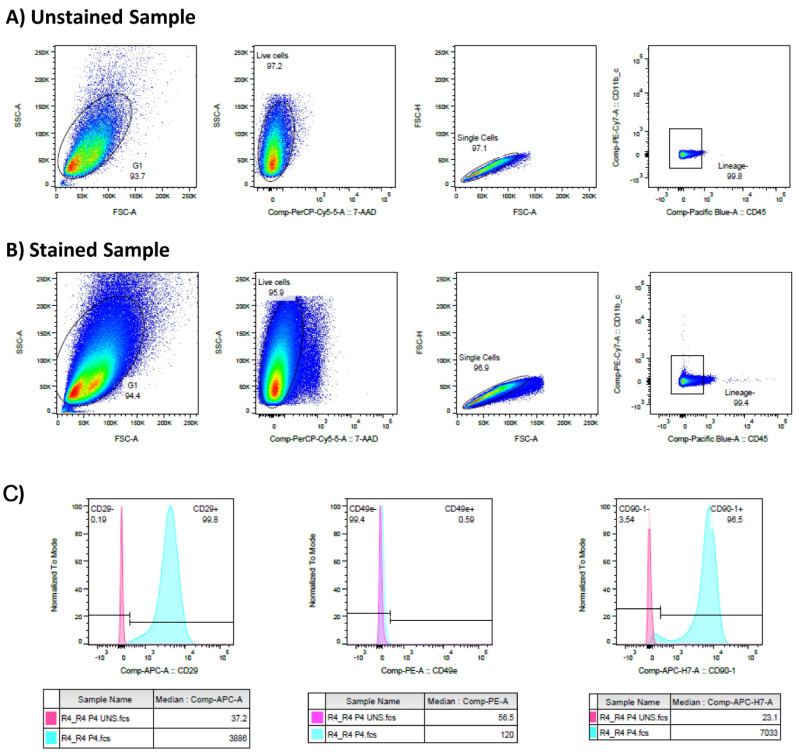
Illustration of the gating strategy used to identify the MSC population in the rat dental pulp isolates. (**A**) The gates were determined in the unstained samples (**B**) and applied to the stained samples. (**C**) The expression levels of CD29, CD49e, and CD90-1 were analyzed as shown in the above histograms, respectively. In total, from six rats, this population of MSCs was identified in 94.58% ± 3.62 of the isolates. From passage two, all the samples already presented with 90% of the population having the characteristics of MSCs (Lineage-CD90-1 + CD29+) and were maintained until passage 10.

The gating strategy used for analyzing the MSC population is illustrated in Figure 5. The percentage of MSCs was calculated from live cells that did not express lineage markers (CD45 and CD11b/c) and moreover expressed CD90 and CD29 simultaneously.

#### 3.1.3. Differentiations Assays

**Figure 6:**
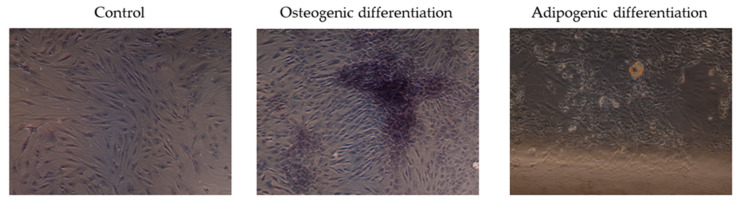
Differentiation ability of DP-MSCs from rats. Control, osteogenic, and adipogenic differentiation. Objective 10×.

After differentiation in the specific media, all DP-MSC samples differentiated into either osteoblasts or adipocytes (Figure 6).

#### 3.1.4. Verifying the Cell Viability of the Implants

Following the surgery, the remains of the vials were seeded on plates. Within a few days, the cells appeared attached to the plate and were therefore viable and proliferating.

### 3.2. Microcomputed Tomography (Micro-CT)

- CONTROL Group

**Figure 7:**
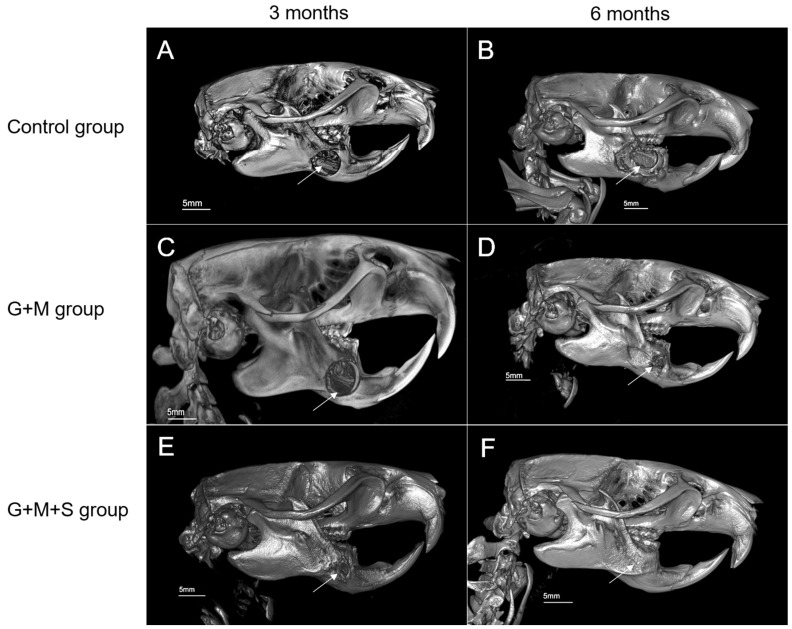
Micro-CT images of defects in different groups at 3 and 6 months: The arrows indicate the site of the bone defect. (**A**) defect in Group C at 3 months, (**B**) defect in Group C at 6 months, (**C**) defect in G + M Group at 3 months, (**D**) defect in G + M Group at 6 months, (**E**) defect in G + M + S Group at 3 months, and (**F**) defect in G + M + S Group at 6 months. Scale bar for Micro-CT images is 5 mm.

Figure 7 shows the defect on the mandible in the Control group, prior to its resection at three (Figure 7A) and six (Figure 7B) months.

- G + M Group

At three months, we observe a significant collapse of the soft tissues and a minor restitution of the defect. The biomaterial has not diminished in volume. The radiopacity of the lesion is highly heterogenous in the center of the defect (Figure 7C).

At six months (Figure 7D), a highlight is the geometric morphology of the defect, which has become smaller, acquiring a denser appearance, significant irregularity in its perimeter, a significantly greater radiological density than in the preceding state, and greater uniformity, although the granulated aspect remained. There is an attempted formation of trabeculae between the biomaterial and the cortical bone, with the hazy appearance of dense bone pathways in the defect treated with the membrane + scaffold, compared to the situation at three months (Figure 7C).

- G + M + S Group

At three months, uniform levels of radiopacity were recorded, with significant consistency in the perimeter and partial structural restoration with new bone (Figure 7E). At six months, there was an almost complete radiological repair of the defects in all cases (80–100%), revealing a high consistency with the bone perimeters and a very uniform distribution of the radiological density (Figure 7F).

### 3.3. Descriptive Histological Analysis

**Figure 8:**
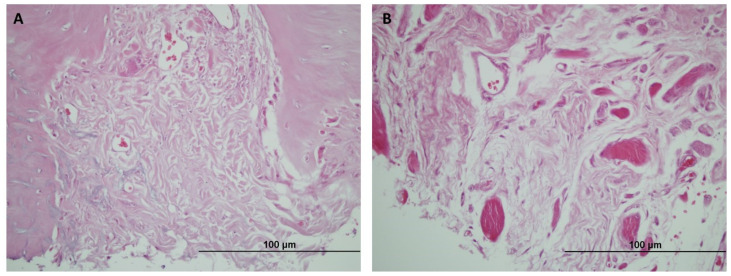
(**A**) Hematoxylin and eosin staining of the bone defect in Group C at 3 months (20× magnification). (**B**) Hematoxylin and eosin staining of the bone defect in Group C at 6 months (40× magnification). The image shows a fibrotic reaction in the untreated lesion. The fibrous cicatricial reaction overran the edges of the lesion. There is no sign of any reactive bone regeneration.

The untreated Control group recorded a fibrous cicatricial reaction, sometimes overrunning the edges of the wound, with no signs of bone regeneration in either of the two timeframes studied (Figure 8).

**Figure 9:**
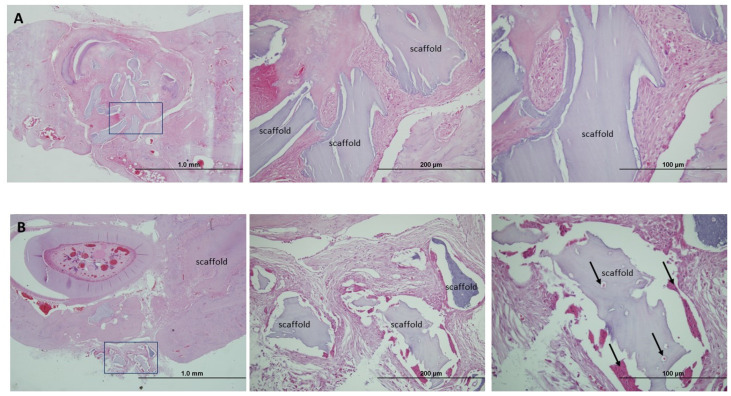
(**A**) Hematoxylin and eosin staining of the bone defect in Group G + M at 3 months (magnifications: 4×, 20×, 40×). Various biomaterial particles and a chronic inflammatory component are visible. (**B**) Hematoxylin and eosin staining of the bone defect in Group G + M + S at 3 months (magnifications: 4×, 20×, 40×). Evidence of osteogenesis is observed. Black arrows indicate osteoblasts embedded in osteoid and signs of neovascularization.

At three months, one of the treated groups, G + M (Figure 9A), showed few signs of bone regeneration, and the edges of the lesion were readily observable. The particles of biomaterial could be seen together with chronic inflammatory infiltration with the presence of multinucleated giant cells (Figure 9A).

For the same timeframe, the other treated group G + M + S (Figure 9B) revealed major cellularity, with a considerable decrease in inflammatory reaction bone-forming activity. We recorded osteoclastogenesis and neoformed bone trabeculae surrounding the biomaterial. There are active osteoblasts and, in some cases, neoformed bone trabeculae around the biomaterial. There are areas with osteoblast precursor cells and osteoblasts partially included in the osteoid, which they are calcifying. There is also abundant immature reticular bone together with osteocytes in the lines of bone development.

**Figure 10:**
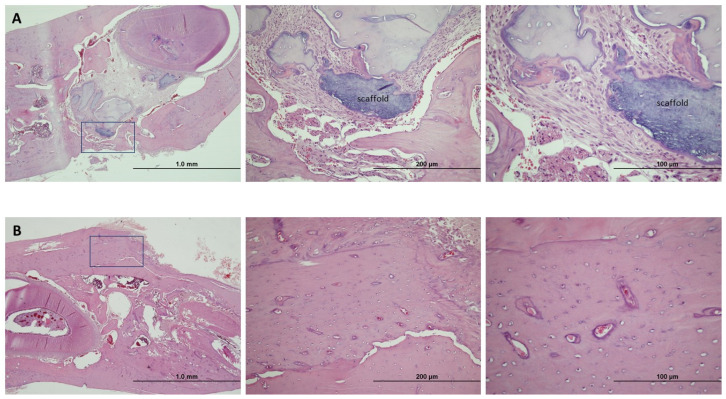
(**A**) Hematoxylin and eosin staining of the bone defect in Group G + M at 6 months (magnifications: 4×, 20×, 40×), showing chronic inflammation. (**B**) Hematoxylin and eosin staining of the bone defect in Group G + M + S at 6 months (magnifications: 4×, 20×, 40×), showing evidence of osteogenesis.

At six months, the treated G + M group continued to have particles of biomaterial encapsulated in a fibrotic matrix. The only signs of new bone were on the edges of the lesion, with a chronic inflammatory component. By contrast, the group treated with DP-MSCs records full bone regeneration with well-organized, vascularized bone with a lamellar structure surrounded by haversian canals. The edges of the lesion are not distinguishable (Figure 10).

### 3.4. Molecular Studies

#### 3.4.1. Endoglin and TFG-β

**Figure 11:**
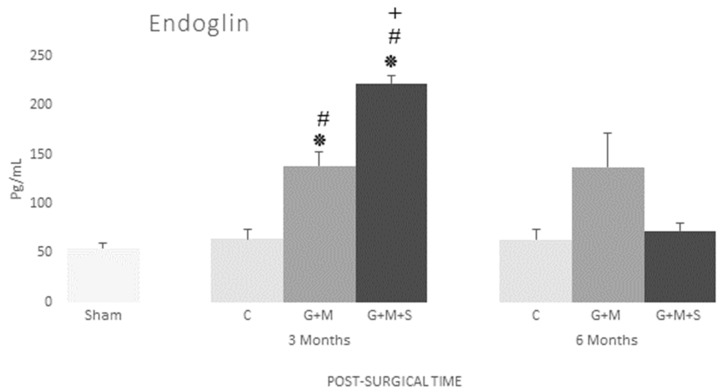
Serum concentration of endoglin (pg/mL) in various groups at 3 and 6 months (Kruskal–Wallis multiple comparison test and Dunn’s test). * *p* < 0.05 compared to the Control group; # *p* < 0.05 compared to the Sham group; + *p* < 0.05 compared to the G + M group.

**Figure 12:**
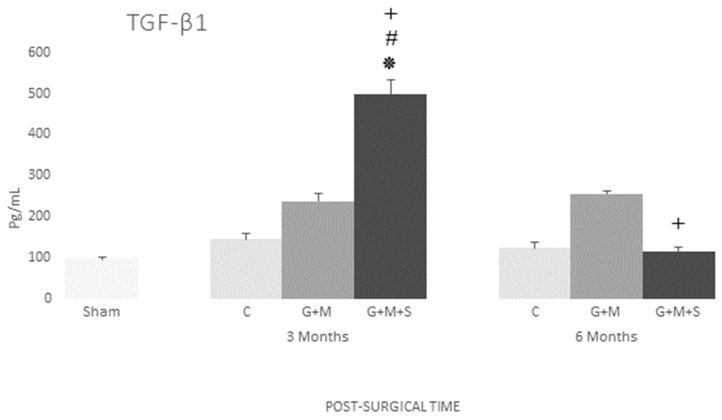
Serum concentration of TGF-β1 (pg/mL) in various groups at three and six months (Kruskal–Wallis multiple comparison test and Dunn’s test). * *p* < 0.05 compared to the Control group; # *p* < 0.05 compared to the Sham group; + *p* < 0.05 compared to the G + M group.

As a neovascularization marker, we studied endoglin, which is crucial for TGF-β1 signaling in endothelial cells. The results for the G + M + S group at three months reveal a significant increase in both endoglin expression and the production of growth factors such as TGFβ1. No changes were found in either the serum levels of endoglin or TGFβ1 in any of the groups at six months (Figure 11 and Figure 12).

#### 3.4.2. Procollagen 1

**Figure 13:**
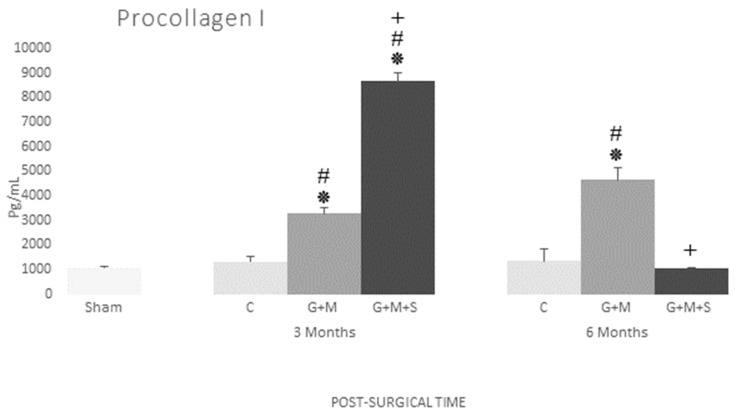
Serum concentration of procollagen (pg/mL) in various groups at three and six months (Kruskal–Wallis multiple comparison test and Dunn’s test). * *p* < 0.05 compared to the Control group; **#** *p* < 0.05 compared to the Sham group; + *p* < 0.05 compared to the G + M group.

The serum levels of procollagen l were higher in the treated groups at three months compared to the Sham and Control groups, with no differences between them. The G + M group at six months continued to record higher serum levels than the Sham and Control groups, while in the G + M + S groups these levels were similar to those in the Sham group (Figure 13).

#### 3.4.3. Parathormone

**Figure 14:**
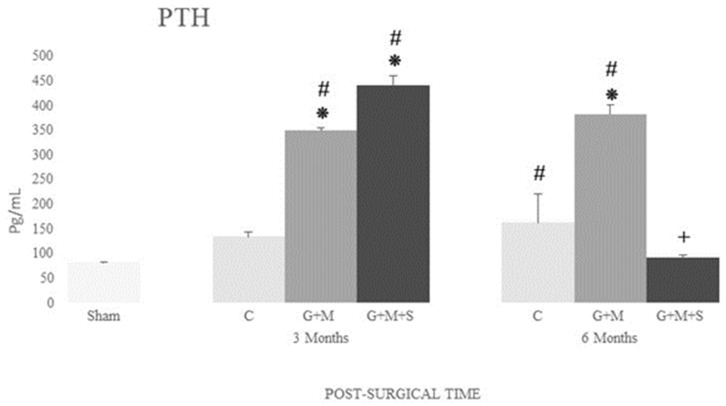
Serum concentration of parathormone (pg/mL) in the various groups at 3 and 6 months (Kruskal–Wallis multiple comparison test and Dunn’s test). * *p* < 0.05 compared to the Control group; **#** *p* < 0.05 compared to the Sham group; + *p* < 0.05 compared to the G + M group.

Following the lesion, we observed higher levels of PTH in all the groups compared to the Sham group, except for the Control group at three months and the G + M + S group at six months. At the same time, the treated groups maintained high serum levels compared to the Control group, except for the G + M + S group at six months (Figure 14).

#### 3.4.4. Calcitonin

**Figure 15:**
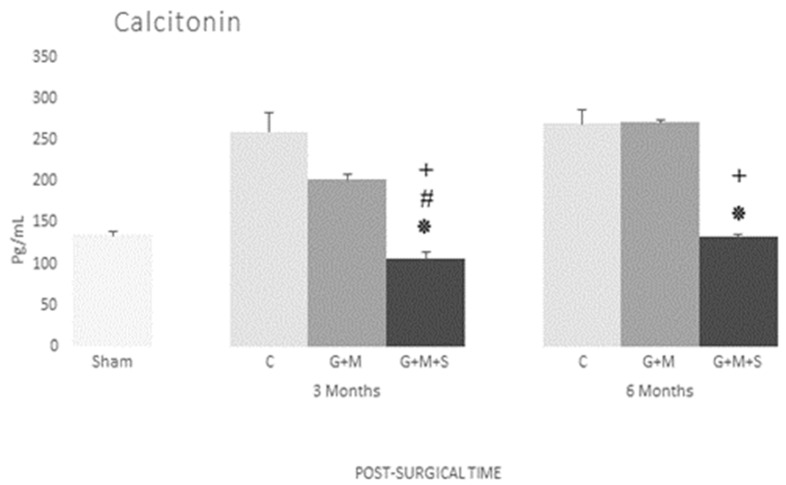
Serum concentration of calcitonin (pg/mL) in various groups at three and six months (Kruskal–Wallis multiple comparison test and Dunn’s test). * *p* < 0.05 compared to the Control group; # *p* < 0.05 compared to the Sham group; + *p* < 0.05 compared to the G + M group.

No changes were found in the serum levels of calcitonin in the Sham, Control, and G + M groups. The G + M + S group, however, recorded a significant decrease at three months, attaining the Sham group’s levels after six months (Figure 15).

## 4. Discussion

An essential step in our assay involved creating a critical bone defect on the mandible that was large enough to avoid spontaneous regeneration. A critical defect is defined as one that regenerates less than 10% of the bone during the animal’s lifetime [25], and in a rat mandible, this will mean an area of more than 4 mm in diameter [26,27,28]. None of the specimens died in the post-operatory period, nor recorded fractures, with a mandibular defect of 5 mm in diameter in all cases. The overall condition of the specimens was good in all the groups, with some swelling of the soft tissue due to the osteotomy, which disappeared spontaneously. The post-surgical clinical monitoring of the specimens treated did not reveal any rejections or degenerative changes due to the dental lesion. The membrane recorded bioresorption, low immunogenicity, stability, and prolonged protection of the covered graft. Our study confirms once more the accessibility of dental pulp for efficiently extracting DP-MSCs. We conclude that the experimental model used is ideal for studying the efficacy of DP-MSCs in osteogenesis, adjudging it to be financially viable with a high performance.

Although the characterization of human derived-MSCs has a well-established criterion, rat MSCs still lack the same consistency, mostly regarding surface markers. Other studies have also reported that some markers, such as CD29 and CD90, were more frequent, while others (e.g., CD73, CD105, CD44, and CD49e) were less so [5,29,30,31,32,33]. Isolates were evaluated here for the presence of CD29, CD90, and CD49e expression and the absence of the expression of the lineage markers CD11b/c and CD45. The presence of the MSC population was confirmed in all the samples tested by the Line-age-CD29 + CD90+ profile. Interestingly, the samples did not express the CD49e marker. These results are consistent with studies conducted by Akpinar et al. [34] (Search for Novel Plasma Membrane Proteins as Potential Biomarkers in Human Mesenchymal Stem Cells Derived from Dental Pulp, Adipose Tissue, Bone Marrow, and Hair Follicle). CD49e is a type I integral membrane glycoprotein known as α5 integrin that associates non-covalently with CD29, a β1 integrin, to form the fibronectin receptor. The relatively low percentage of the CD49e+ population, ranging from 1 to 30%, might be explained by the sensitivity of the integrin α5β1 to trypsin [35,36]. The use of this proteolytic enzyme could therefore alter the antibody binding site, diminishing its recognition by the clone of the antibody used. Nevertheless, the expression of CD90 and CD29 and the absence of lineage markers can identify the MSC population.

The results from the radiological and histological studies show no evidence of osteogenic activity in either Group C or in the G + M group, confirming that these conditions require enough cell material and a high osteogenic capability. A major characteristic of DP-MSCs that makes them a promising osteogenic candidate is their co-differentiation in osteoblasts and endotheliocytes, fully integrating the blood vessels within the ossification lines, prompting the formation of vascularized bone tissue [37]. A clinical trial conducted in 2019 [38] found that these cells produce extracellular vesicles (EVs) that contain pro-angiogenic factors (e.g., VEGF, angiopoietin, or angiogenin). The Gen-Os^®^ biomaterial’s angiogenic potential is also greater than other synthetic materials, as it favors the secretion of VEGF [39,40].

DP-MSCs are therefore the mainstay of the osteogenic process ending at six months with full bone formation. DP-MSCs contain immunomodulating and anti-inflammatory factors such as IL-6, IL-8, TGF-β1, hepatocyte growth factor (HGF), and indoleamine 2,3-dioxygenase (IDO) [41,42], all of which play an important part in regulating the equilibrium between the anti- and pro-inflammatory properties of cytokines [42,43,44], suppressing the activation of T cells, the proliferation of peripheral blood mononuclear cells, and even allogeneic immune responses [41,45].

In this study, the group treated with DP-MSCs at three months, like TGF-β1, recorded high levels of endoglin expression, with this protein being part of the receptor complex of TGF-β1 that is highly expressed in several kinds of proliferating cells and stem cells in differentiation during the initial stages of healing. Angiogenesis is a major step in bone healing, and endoglin is highly expressed in proliferating endothelial cells and pericytes during angiogenesis. This means it is not only a proliferation marker, as it also regulates cell functions such as adhesion, migration, and cell permeability. Endoglin has a significant role to play in the regulation of the movement of stem cells from a specific niche to the damaged region [46]. Besides high levels of TGF-β1, our results have revealed an increase in collagen I content in the G + M + S group at three months, confirming that the TGF-β1 factor is involved in cell differentiation and collagen synthesis [47]. As type I collagen is the main component of the organic matrix, this increase in our results will render the bone tissue flexible and resistant to impact loads [37].

TGF-β1 regulates the proliferation and differentiation of chondrocytes and osteoblasts [48,49], although its osteoinductive properties mainly render it a major growth factor, performing a key role in the different stages of the bone healing process [50] by regulating the production of the extracellular matrix (ECM), proteases, protease inhibitors, migration, chemotaxis, and the proliferation of different kinds of cells, including stem cells, which regulate angiogenesis and the formation of granulation tissue, remodeling of the matrix, and scar maturation.

The implementation of DP-MSCs on the biomaterials also increases the serum levels of PTH at three months, favoring bone regeneration, as it has osteoclastic and osteogenic activity, whereby the bone formation process promoted by the PTH is very similar to the same process occurring under physiological conditions. Bone repair is not a unique osteogenic process and requires angiogenesis and the participation of osteoclasts [51]. Bone repair without osteoclastic activity may lead to excessive bone development [52]. PTH regulates the differentiation pathway of bone marrow mesenchymal stem/stromal cells (BM-MSCs), increases their osteogenic differentiation, and inhibits their adipogenic differentiation [53,54].

This study has also found a reduction in the serum concentration of CT in the group treated with DP-MSCs. The changes observed in the levels of calciotropic hormones in the rats could increase bone resorption and promote mineral metabolism in the DP-MSC group [55].

The alloplastic matrices take a backseat as the mere facilitator of cell functions, although it is essential to use the most appropriate ones to provide cell support and facilitate regeneration with osteoinductive factors. Each bone substitute has different properties, and Gen-Os^®^ (OsteoBiol^®^, Italy) appears to provide the best guarantees for osteogenesis. Indeed, an assay [56] shows that the proliferation and recruitment of MSCs is higher with Gen-Os^®^ (OsteoBiol^®^, Italy) than with other alloplast grafts such as Bio-Oss^®^. It also produces a smaller inflammatory reaction than other synthetic biomaterials [57].

It seems fair to say that the properties of the different elements used together favor osteogenesis, acting as an ideal bone substitute and enabling the bone to regenerate while upholding all its characteristics.

## 5. Conclusions

The findings of our study on the combination of DP-MSCs, Gen-Os^®^, and Evolution^®^ (OsteoBiol^®^, Italy) reveal an almost complete structural restitution of the defect and full regeneration with well-organized and vascularized bone. This recovery is explained by an environment of bone regeneration and proliferation induced by increases in endoglin, TGF-β1, protocollagen, parathormone, and calcitonin.

The cell therapy associated with the high concentration of osteoinductive factors in the biomaterial could become the ideal combination for treating bone defects. Nonetheless, further research is required on action mechanisms, therapeutic dosages, and other possible variables for validating these results.

## Data Availability

The raw data supporting the conclusions of this article will be made available by the authors on request.
